# Regular exercise and branched‐chain amino acids prevent ischemic acute kidney injury‐related muscle wasting in mice

**DOI:** 10.14814/phy2.14557

**Published:** 2020-08-26

**Authors:** Soichiro Nagata, Akihiko Kato, Shinsuke Isobe, Tomoyuki Fujikura, Naro Ohashi, Hiroaki Miyajima, Hideo Yasuda

**Affiliations:** ^1^ Internal Medicine 1 Hamamatsu University School of Medicine Hamamatsu Japan; ^2^ Blood Purification Unit Hamamatsu University Hospital Hamamatsu Japan

**Keywords:** acute kidney injury, branched‐chain amino acid, exercise, muscle wasting

## Abstract

Acute kidney injury (AKI) causes glucose and protein metabolism abnormalities that result in muscle wasting, thereby affecting the long‐term prognosis of critical illness survivors. Here, we examined whether early intervention with treadmill exercise and branched‐chain amino acids (BCAA) can prevent AKI‐related muscle wasting and reduced physical performance in mice. Unilateral 15 min ischemia‐reperfusion injury was induced in contralateral nephrectomized mice, and muscle histological and physiological changes were assessed and compared with those of pair‐fed control mice, since AKI causes severe anorexia. Mice exercised for 30 min each day and received oral BCAA for 7 days after AKI insult. By day 7, ischemic AKI significantly decreased wet weight, myofiber cross‐sectional area, and central mitochondrial volume density of the anterior tibialis muscle, and significantly reduced maximal exercise time. Regular exercise and BCAA prevented AKI‐related muscle wasting and low physical performance by suppressing myostatin and atrogin‐1 mRNA upregulation, and restoring reduced phosphorylated Akt and PGC‐1α mRNA expression in the muscle. Ischemic AKI induces muscle wasting by accelerating muscle protein degradation and reducing protein synthesis; however, we found that regular exercise and BCAA prevented AKI‐related muscle wasting without worsening kidney damage, suggesting that early rehabilitation with nutritional support could prevent AKI‐related muscle wasting.

## Graphical abstract

The combination of regular exercise and BCAA supplementation for 7 days prevented AKI‐related muscle wasting by suppressing myostatin and atrogin‐1 mRNA upregulation, and restoring Akt phosphorylation and PGC‐1α mRNA expression without causing further kidney dysfunction.

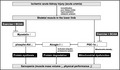

## 1 INTRODUCTION

Sarcopenia and frailty are components of geriatric syndrome that are currently regarded as important medical and social challenges. Not only are these syndromes caused by aging, but also by disuse, malnutrition, and systemic diseases such as chronic kidney disease (CKD). Approximately 20%–34% of non‐dialysis‐dependent CKD patients and 70% of dialysis‐dependent CKD patients suffer from either sarcopenia or frailty, which are associated with poor renal and survival outcomes, hospitalization, low quality of life, and costly healthcare (Janssen, Shepard, Katzmarzyk, & Roubenoff, 2004; Johansen, Chertow, Jin, & Kutner, 2007; Lacson et al., 2010; McAdams‐DeMarco et al., 2015; Robinson, Wu, Stiegmann, & Moss, 2011; Roshanravan et al., 2012).

Acute kidney injury (AKI) often develops following severe sepsis, invasive cardiovascular surgery, and exposure to nephrotoxic agents in hospitals. Despite the development of renal replacement therapy, the prognosis of AKI remains poor and the condition incurs high medical costs (Chertow, Burdick, Honour, Bonventre, & Bates, 2005; Hoste et al., 2006). Small and transient increases in serum creatinine (Scr) levels are associated with a lower 10‐year survival rate (Linder et al., 2014), suggesting that even mild AKI can affect long‐term survival (Doyle & Forni, 2016; Grams & Rabb, 2012). Recently, it has been shown that AKI is associated with the development of muscle wasting and frailty in survivors of critical illness (Abdel‐Kader et al., 2018), and is closely related to the development of frailty, particularly in the elderly (Jiesisibieke et al., 2019). AKI is also a robust predictor of unplanned hospital readmission in critical care patients who survive following hospitalization (Horkan et al., 2015); thus, AKI‐related muscle wasting and frailty are strongly associated with post‐discharge outcomes.

In older adults, the primary strategy for preventing sarcopenia and frailty is regular exercise and sufficient intake of dietary protein enriched with branched‐chain amino acids (BCAA; leucine, isoleucine, valine) (Liao et al., 2017), which have been found to effectively prevent sarcopenia in CKD patients without accelerating kidney dysfunction (Castaneda et al., 2001; Manfredini et al., 2017; Watson et al., 2018). In addition, early rehabilitation twice per day for 30 min during the first week of septic shock has been shown to prevent declining myofiber cross‐sectional area (CSA) (Hickmann et al., 2018). Although oral BCAA supplementation alone was unable to ameliorate muscle wasting in CKD rats with 5/6 nephrectomy (Yoshida et al., 2017), no studies have yet examined whether early intervention with combined exercise and BCAA supplementation could prevent AKI‐related muscle wasting, or have elucidated the associated mechanisms.

This study sought to explore the mechanisms of skeletal muscle wasting following renal ischemia‐reperfusion injury (IRI). Since AKI causes anorexia, we evaluated alterations in muscle protein synthesis and degradation markers under strict pair feeding before examining the effect of regular exercise and BCAA supplementation for 7 days on functional and morphological changes, muscle proteins, and degradation signaling.

## 2 MATERIALS AND METHODS

### 2.1 Animal preparation

All protocols were approved by the ethics review committee for animal experimentation of Hamamatsu University School of Medicine (approval number: 2,017,068). Ten‐week‐old C57BL/6J male mice were purchased from SLC Japan and housed under standard laboratory conditions (temperature: 24 ± 2°C, humidity: 55 ± 5%, light/dark cycle: 12/12 hr). The animals were permitted to move freely within their cages and provided with clean drinking water and dietary chow ad libitum prior to the experiments.

### 2.2 Induction of ischemic AKI

**Figure 1:**
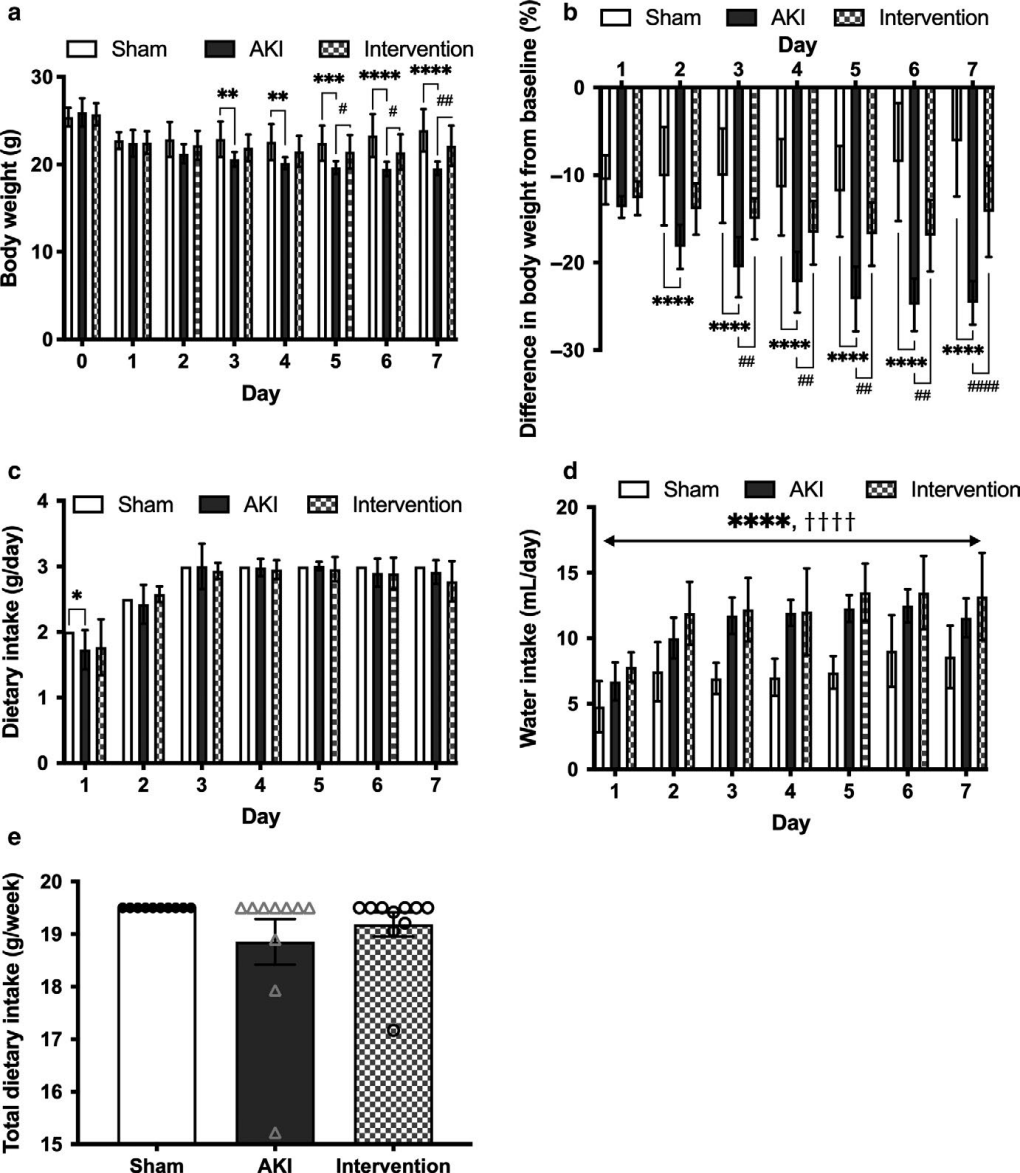
Experimental protocols. AKI, acute kidney injury; BCAA, branched‐chain amino acids; and IRI, ischemia‐reperfusion injury

After 7 days of acclimation, mice were subjected to unilateral right nephrectomy under anesthesia with 5% of sevoflurane, returned to individual cages, and divided into three groups: sham‐operated group (right nephrectomy + sham IRI), AKI group (right nephrectomy + renal IRI), and AKI intervention group (right nephrectomy + ischemic AKI + exercise and BCAA treatment) (Figure 1). Briefly, mice were intraperitoneally administered with three anesthetics (medetomidine hydrochloride, 0.3 mg/kg body weight (BW), midazolam 4 mg/kg BW, and butorphanol tartrate 5 mg/kg BW) after which either the sham operation or left renal artery clamping was performed. The left renal hilum was clamped for 15 min using a cerebral aneurysm clip and kidney color was confirmed to return from black to red upon its release. After skin closure, mice were woken using atipamezole hydrochloride (0.3 mg/kg BW) and administered with 0.2 ml of 0.9% saline for body fluid deprivation and 0.25 mg/kg BW of buprenorphine as an analgesic. During surgery, rectal temperature was maintained at 37–38°C using a heat pad and infrared lamp.

BW, dietary chow, and drinking water intake were assessed daily for 7 days. Since AKI mice ate less than sham‐operated mice (mean difference: −2.0 g, day 0 to 1; −1.5 g, day 1 to 2; −1.0 g, day 2 to 7), all groups were given the same amount of chow to avoid confounding effects. Mice were euthanized by sevoflurane inhalation after which blood, tibialis anterior muscle, and kidney samples were collected on days 1 (*n* = 10 per group) and 7 (*n* = 10 per group).

### 2.3 Exercise training and BCAA supplementation

The treadmill‐training program used by TMS‐4BA (MELQUEST) was preceded by a 7‐day adaptation period consisting of 5 min increments at 10 m/min. After AKI induction, mice exercised for 30 min per day for 7 days, with intensity increased according to the following protocol: 10° at 9.6 m/min for the first 2 days and 10° at 12.8 m/min thereafter (Uchiyama, Jin, Yin, Shimokawa, & Niimi, 2015). In preliminary study, this was the maximum load, which the mice with AKI could run. Drinking water for each cage was prepared with 1.0% BCAA (leucine:isoleucine:valine, 3:1:1) on day 1 and 0.6% BCAA thereafter, as described previously (Baptista et al., 2013; Yoshida et al., 2017).

### 2.4 Assessment of changes in muscle weight and histology

The tibialis anterior muscles of both legs were removed immediately after euthanasia and their mean wet weight calculated. Muscle samples were taken from one‐third of the distance along the peripheral side, fixed with 4% of paraformaldehyde at 25 ± 3°C for 24 hr, and embedded in paraffin. The samples were then cut into 10 μm thick sections, stained with hematoxylin and eosin. The CSA of 200 myofibers per sample was measured using ImageJ software (National Institutes of Health) and reported as the mean (Yoshida et al., 2017).

### 2.5 TEM analysis of mitochondrial morphometry

Muscle tissue samples were fixed with 2% of glutaraldehyde at 4°C for 2 hr, embedded in Quetol 812, and cut into ultra‐thin sections for TEM (JEM‐1220, JEOL). Two areas were randomly selected per sample and 40 images taken (final magnification, 20,000×). Interfibrillar mitochondrial volume density was calculated using the point counting method (Hoppeler et al., 1985; Hoppeler, Lüthi, Claassen, Weibel, & Howald, 1973).

### 2.6 Exercise tolerance test

Running time was measured on day 7 following AKI (*n* = 7 per group) as described previously (Enoki et al., 2017). Three days earlier, mice were acclimated to a motor‐driven treadmill at 10 m/min with a 0% incline for 5 min. To assess walking ability, mice were forced to run according to the protocol described in Table S2 until they were fully exhausted (i.e., remained on the electrical shocker plate for >30 s). Electrical stimulation was set at the intensity recommended by the manufacturer.

### 2.7 Measurement of tGFR

To measure tGFR non‐invasively, mice were lightly anesthetized with 5% sevoflurane, a monitoring device (Medibeacon) placed on their back, and fluorescein‐isothiocyanate (FITC)‐sinistrin (50 mg/kg, Medibeacon) injected into their retro‐orbital sinus. After 1.5 hr, the device was removed and transcutaneous GFR (µl/min) was estimated by analyzing changes in the plasma half‐life of FITC‐sinistrin using the three‐compartment model according to the manufacturer's instructions.

### 2.8 Measurement of BUN

Blood samples were collected and spun in a 4°C centrifuge at 4,500 rpm. Plasma urea nitrogen levels were determined using enzymatic assays (Sanritsu Zelkova).

### 2.9 Renal histological analysis

The left kidney was fixed in 4% of paraformaldehyde for 24 hr, embedded in paraffin, and cut into 3 μm sections that were then stained with periodic acid Schiff reagent. At 400× magnification, 15 areas were randomly selected in the outer stripe of the outer medulla and the severity of tubular damage in the proximal tubule was scored as follows: 0, no damage; 1, <25% area damaged; 2, 25%–50%; 3, 50%–75%; 4, >75%, and calculated as the average per kidney (Wei et al., 2010).

### 2.10 RNA extraction, reverse transcription, and real‐time quantitative RT‐PCR

The left tibialis anterior muscle was placed in RNAlater Stabilization Solution (Invitrogen) at 4°C for 24 hr and then stored at −80°C until further analysis. RNA was extracted from muscle tissue samples using an RNeasy Plus Universal Mini Kit (Qiagen) and homogenized using a bead‐type crusher (MS‐100R, Tomy Seiko). RNA concentration was measured using a Nano Pad (DeNovix), and 1 μg of total RNA was reverse transcribed into cDNA using a high capacity cDNA RT kit with an RNase inhibitor (Applied Biosystems) according to the manufacturer's instructions. qRT‐PCR was performed using Power SYBR^®^ Green PCR Master Mix (Applied Biosystems) and a StepOnePlus Real‐Time PCR System (Applied Biosystems). Target mRNA abundance was normalized to β‐actin and quantified as a ratio of the mean value from sham‐operated mice using the 2^−ΔΔCT^ method (Livak & Schmittgen, 2001). The primer sequences used are shown in Table S1.

### 2.11 Western blotting analysis

Right tibialis anterior muscle samples were stored at −80°C before being dissolved in 10× cell lysis buffer (Cell Signaling Technology) and 1 nM of phenylmethylsulfonyl fluoride. Samples were homogenized using a bead‐type crusher (MS‐100R, Tomy Seiko) and protein concentration measured using a protein assay kit (Micro TP‐Test, WAKO) according to manufacturer's instructions. Samples were adjusted to the same protein concentration, boiled at 95°C for 5 min, and then 30 μg subjected to 10% of sodium dodecyl sulfate–poly‐acrylamide gel electrophoresis. Samples were transferred to polyvinylidene difluoride membranes using NuPAGE transfer buffer and antioxidant (Invitrogen). The membranes were blocked with 5% of bovine serum albumin for 1 hr at 25 ± 3°C on a horizontal shaker and incubated overnight with the following primary antibodies at 4°C: p‐Akt (Ser473; #9271, 1:2,000, Cell Signaling Technology), Akt (#9272, 1:5,000, Cell Signaling Technology), LC3A/B (D3U4C; #12741, 1:2,000, Cell Signaling Technology), and GAPDH (#sc‐47724, 1:50,000, Santa Cruz Biotechnology). The membranes were then incubated with anti‐rabbit immunoglobulin G (IgG) horseradish peroxidase (HRP)‐conjugated antibodies (#W4011, 1:5,000, Promega) or anti‐mouse IgG HRP‐conjugated antibodies (#sc‐2357, 1:5,000, Santa Cruz Biotechnology) for 40 min at 25 ± 3°C. Bands were detected using a Fusion system (Vilber Lourmat). Band intensity was quantified using ImageJ software (NIH).

### 2.12 Statistical analysis

Data were expressed as mean ± *SD*. Differences among groups were analyzed by one‐ or two‐way ANOVA analysis of variance. Between‐group comparisons were analyzed by Tukey's multiple comparison test. All analyses were conducted in GraphPad Prism 8 (GraphPad Software). *p* < .05 were considered significant.

## 3 RESULTS

### 3.1 Changes in dietary intake, body weight (BW), muscle morphology, and muscle function following AKI

**Figure 2:**
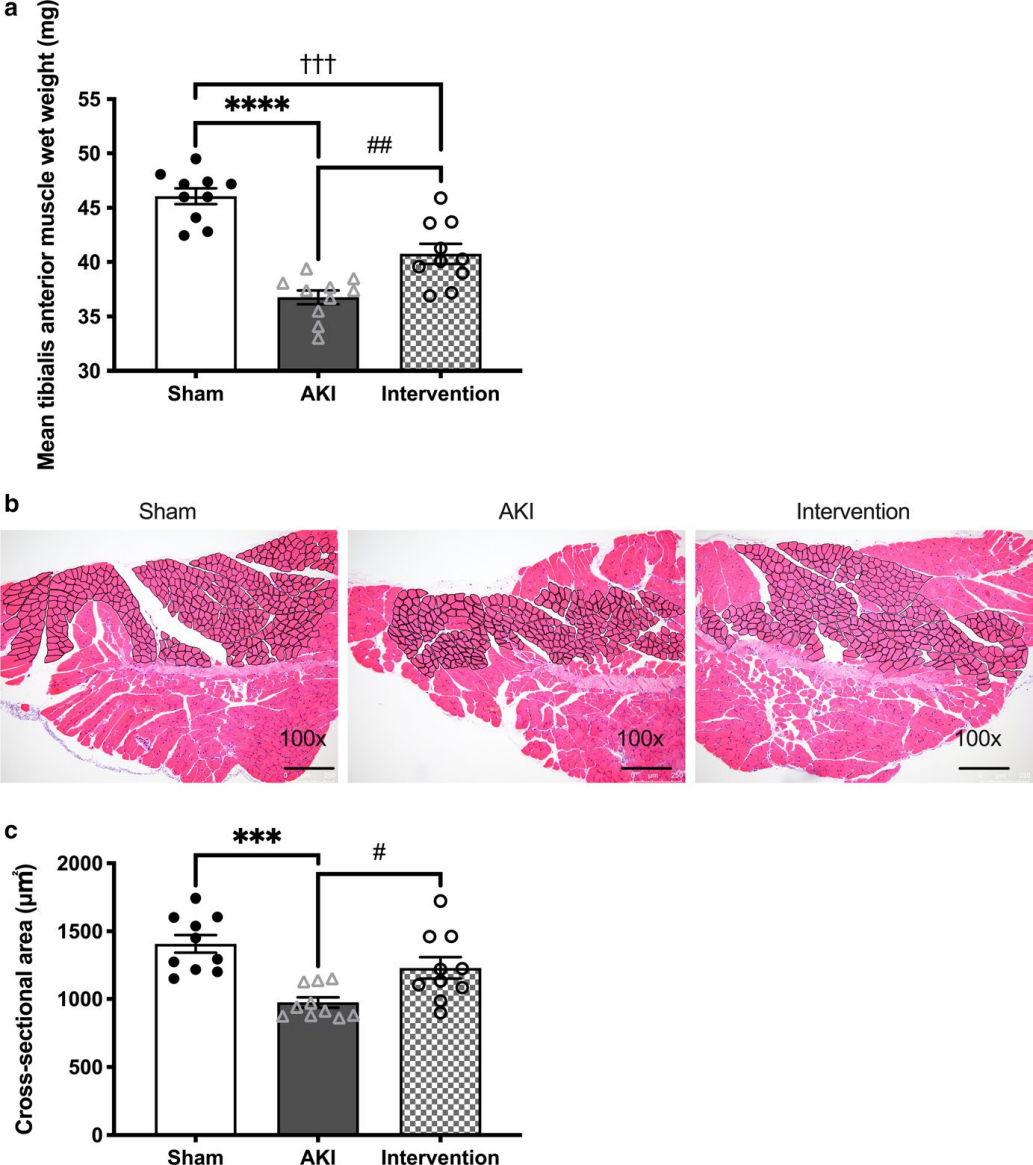
Effect of AKI on anorexia. Serial changes in body weight (a), percentile decline of body weight from day 0 (b), dairy chow intake (c), daily drinking water intake (d), and total dietary intake for 7 d (e). Data represent the mean ± *SD* (*n* = 10 per group). Significant differences between the sham‐operated and AKI groups: *p < .05, **p < .01, ***p < .001, ****p < .0001. Significant differences between the AKI and AKI intervention groups: #*p* < .05, ##*p* < .01, ####*p* < .0001. Significant differences between sham‐operated and AKI intervention groups: †††*p* < .001, ††††*p* < .0001. AKI, acute kidney injury

**Figure 3:**
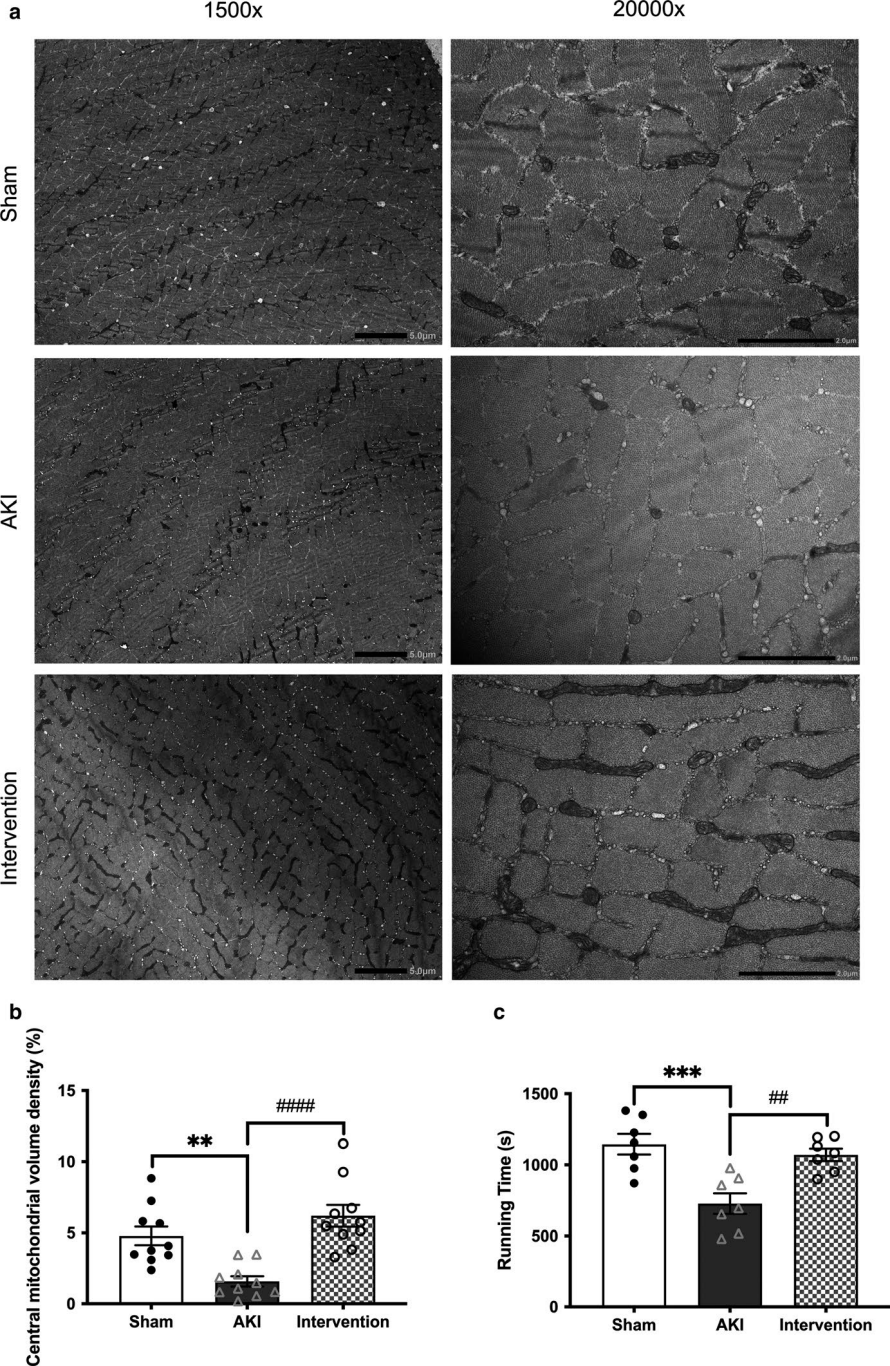
Effect of combined exercise and BCAA supplementation on muscle. Wet weight of tibialis anterior muscle (a), muscle histology at 100× magnification (b), and mean cross‐sectional area of 200 myofibers (μm^2^) 7 d after ischemic AKI. Black bar represents 250 μm width at 100*×* magnification. Data represent the mean ± *SD* (*n* = 10 per group). Significant differences between the sham‐operated and AKI groups: ***p < .001, ****p < .0001. Significant differences between the AKI and AKI groups: #*p* < .05, ##*p* < .01. Significant differences between the sham‐operated and AKI intervention groups: †††*p* < .001. AKI, acute kidney injury

**Figure 4:**
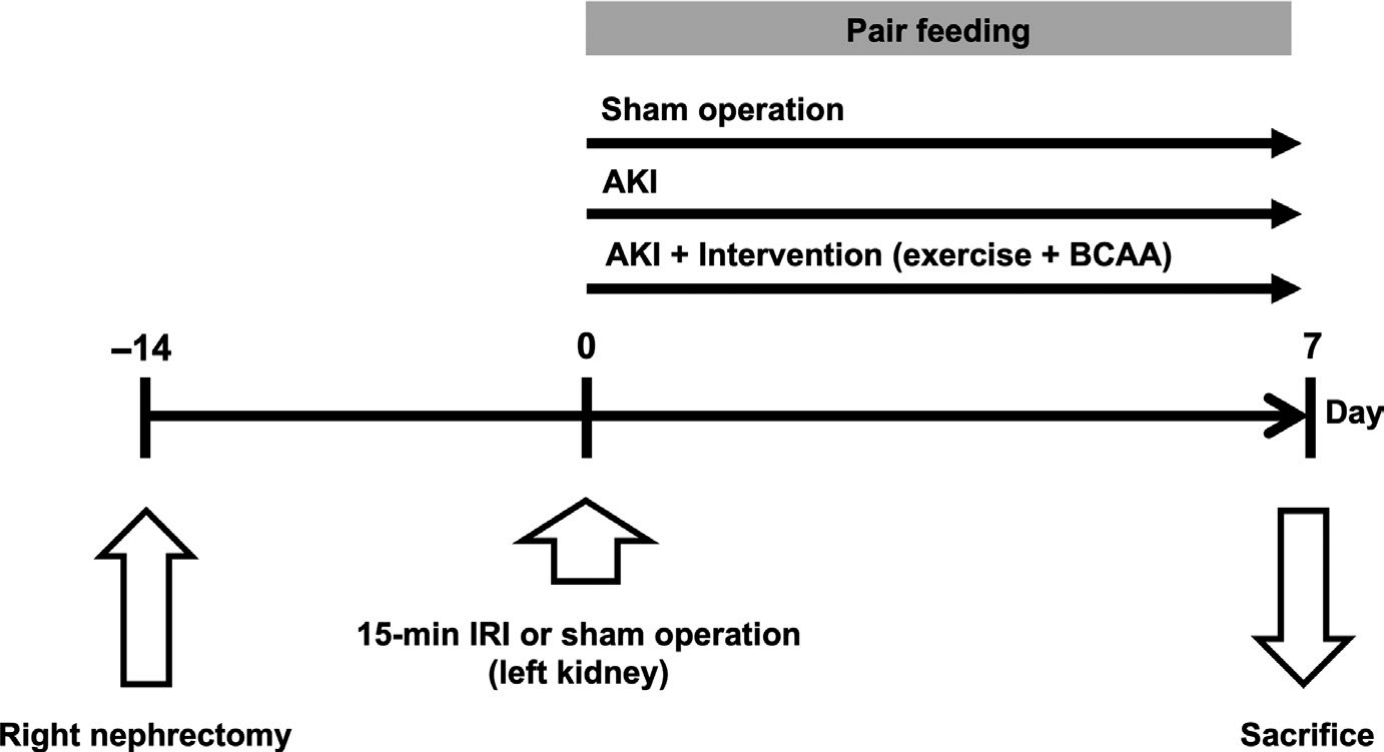
Effect of combined exercise and BCAA supplementation on mitochondria biogenesis. Comparison of transmission electron microscopy (a), central mitochondrial volume density (b), and exercise tolerable time (c). Lower interfibrillar mitochondria density in muscle sections from AKI mice compared to sham‐operated mice (b). Exercise tolerable time was significantly shorter in the AKI group than in the sham‐operated group (c). Black bar represents 5.0 μm width at 1,500*×* and 2.0 μm width at 20,000*×* magnification (a). Data represent the mean ± *SD* (*n* = 10 (b) and 7 (c) per group). Significant differences between the sham‐operated and AKI groups. **p < .01, ***p < .001. Significant differences between the AKI and AKI intervention groups: ##*p* < .01, ####*p* < .0001. AKI, acute kidney injury

There were no significant differences observed in basal BW between the AKI and sham‐operated mice (25.9 ± 1.62 versus 25.4 ± 1.06 g); however, a greater decrease in BW was noted in the AKI group compared to the sham‐operated group by day 7, despite pair feeding (Figure 2a–c). The wet weight of the tibialis anterior muscle (*p* < .0001, Figure 3a) and myofiber CSA (*p* < .001, Figure 3b,c) also decreased more significantly in the AKI mice. Transmission electron microscopy (TEM; 20,000× magnification) revealed that the density of interfibrillar mitochondria was significantly lower in the muscles of AKI mice (Figure 4a,b), while the maximal tolerable exercise time was significantly shorter in the AKI group compared to the sham‐operated group (727 ± 193 versus 1,145 ± 190 s, *p* < .001; Figure 4c). We, therefore, confirmed that ischemic AKI caused muscle wasting despite the strict pair feeding.

### 3.2 Effect of exercise and BCAA supplementation on muscle morphology and function

Daily dietary chow and drinking water consumption were identical between the AKI groups with and without intervention (Figure 2c,d) and no difference occurred in total chow intake over 7 days between the two groups (18.9 ± 1.37 versus 19.2 ± 0.72 g/week, *p* = .69; Figure 2e). The estimated energy intake from BCAA was 0.24–0.30 kcal/day, which is much lower than that obtained from standard chow (6.6–9.9 kcal/day). Furthermore, combined treadmill exercise and oral BCAA supplementation for 7 days significantly increased tibialis anterior muscle weight (36.8 ± 2.01 versus 40.8 ± 2.91 mg, *p* < .0001, Figure 33a), myofiber CSA (975 ± 118 versus 1,230 ± 251 μm^2^, *p* < .05, Figure 3b,c), interfibrillar mitochondrial volume density (1.59 ± 1.14 versus 6.19 ± 2.43%, *p* < .0001, Figure 4b), and running time (727 ± 193 versus 1,071 ± 116 s, *p* < .01, Figure 4c). These findings suggest that regular exercise combined with BCAA supplementation for 7 days mitigated AKI‐related muscle wasting.

### 3.3 Effect of exercise and BCAA supplementation on ischemic AKI severity

**Figure 5:**
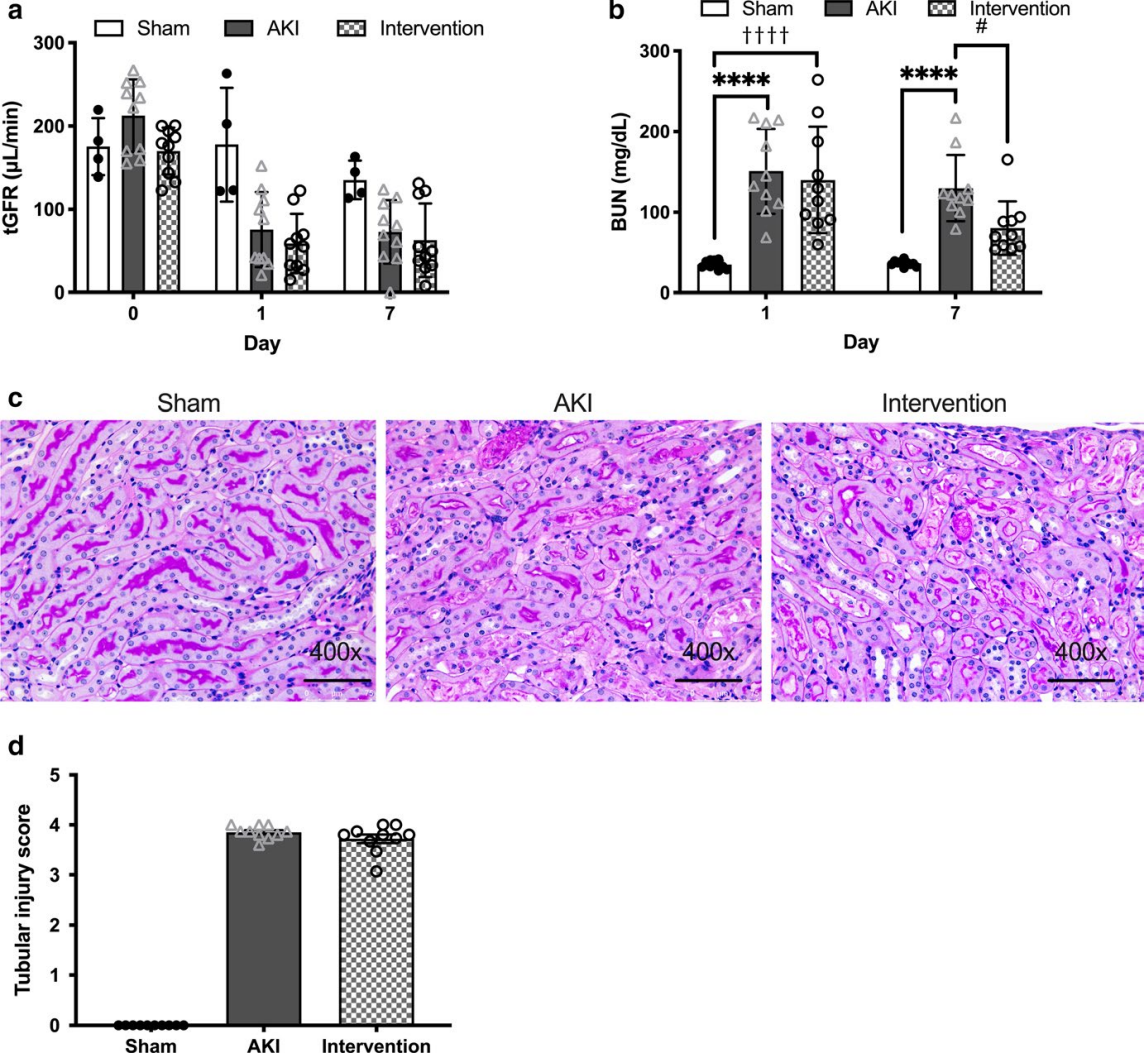
Effect of combined exercise and BCAA supplementation on AKI severity. BUN was significantly lower in the AKI intervention group (a) but no differences in transcutaneous GFR (b) or tubular damage were observed between the three groups (c,d). Black bar represents 75 μm width at 400*×* magnification. Data represent the mean ± *SD* (*n* = 10 per group). Significant differences between the sham‐operated and AKI groups: ***p < .001, ****p < .0001. Significant differences between the AKI and AKI intervention groups: #*p* < .05. Significant differences between the sham‐operated and AKI intervention groups: ††††*p* < .0001. AKI, acute kidney injury

AKI mice displayed significantly lower transcutaneous glomerular filtration rates (tGFR) and higher blood urea nitrogen (BUN) levels on days 1 and 7 (Figure 5a,b), with tubular necrosis observed in 5%–30% of the proximal tubules (Figure 5c,d). Although the combined treatment significantly decreased BUN, it had no effect on tGFR or the tubular damage score on day 7 (Figure 5a–d). Thus, the combined exercise and BCAA treatment did not exacerbate the AKI‐related renal damage over 7 days.

### 3.4 Changes in muscle‐related molecular markers following AKI

**Figure 6:**
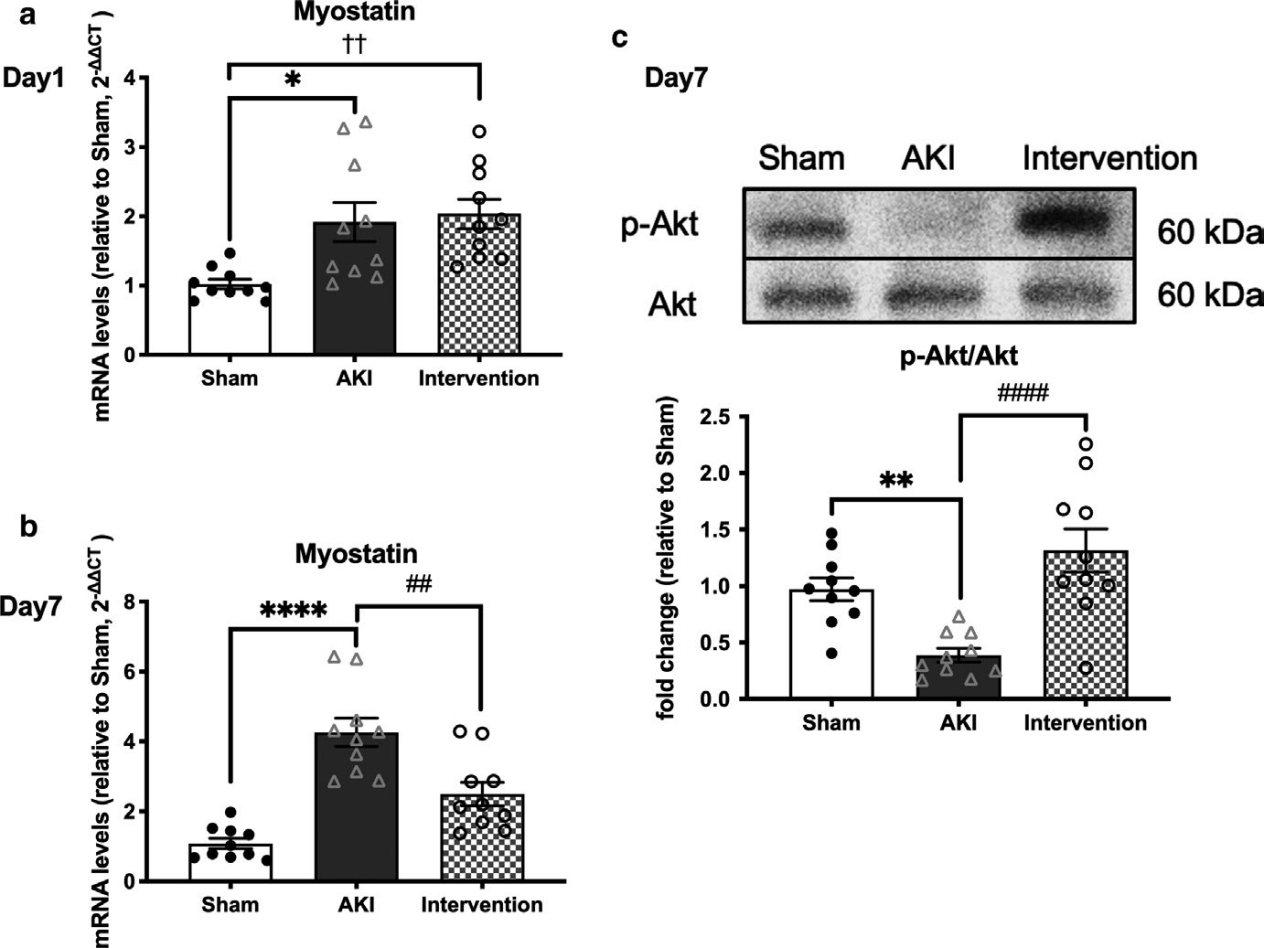
Muscle myostatin mRNA expression and Akt phosphorylation in AKI. AKI significantly increased myostatin mRNA expression on days 1 (a) and 7 (b) after ischemic AKI. Regular exercise and BCAA supplementation prevented AKI‐induced myostatin mRNA upregulation on day 7. AKI decreased the p‐Akt to Akt ratio in muscle at day 7 while treatment restored the ratio to baseline levels (c). Data represent the mean ± *SD* (*n* = 10 per group). Significant differences between the sham‐operated and AKI groups: *p < .05, **p < .01, ****p < .0001. Significant differences between the AKI and AKI intervention groups: ##*p* < .01, ####*p* < .0001. Significant differences between the sham‐operated and AKI intervention groups: ††*p* < .01, ††††*p* < .0001. AKI, acute kidney injury

**Figure 7:**
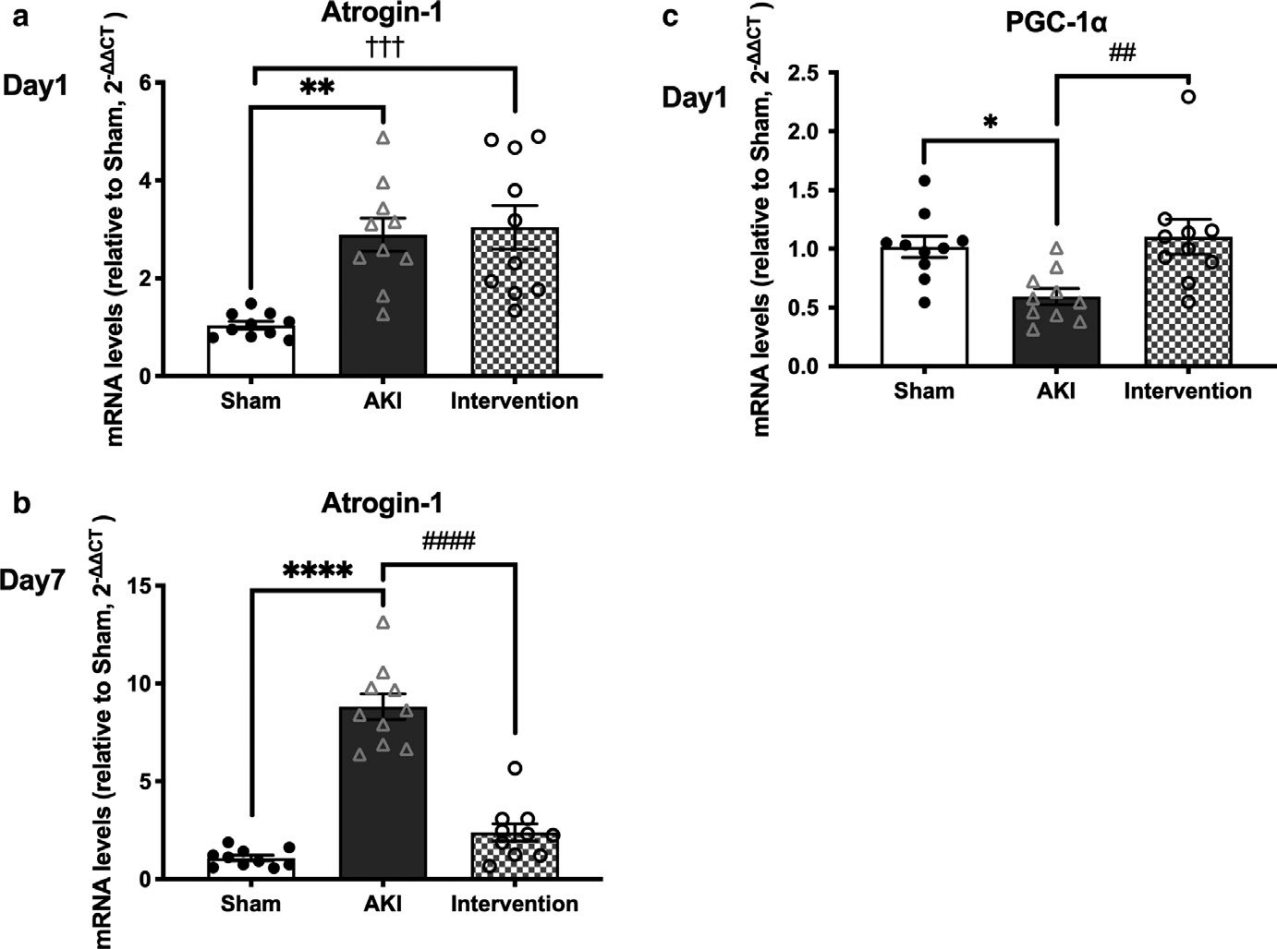
Changes in muscle atrogin‐1 and PGC‐1α mRNA expression. AKI significantly increased atrogin‐1 mRNA expression on days 1 (a) and 7 (b) and decreased PGC‐1α mRNA expression. Combined therapy decreased AKI‐induced increases in atrogin‐1 mRNA on day 7 and PGC‐1α mRNA on day 1. Data represent the mean ± *SD* (*n* = 10 per group). Significant differences between the sham‐operated and AKI groups: *p < .05, **p < .01, ****p < .0001. Significant differences between the AKI and AKI intervention groups: ##*p* < .01, ###*p* < .001. Significant differences between the sham‐operated and AKI intervention groups: †††*p* < .001. AKI, acute kidney injury. PGC‐1α, peroxisome proliferator‐activated receptor gamma coactivator 1α

In the AKI group, myostatin mRNA expression was significantly upregulated by 1.92‐fold on day 1 (*p* < .05) and 4.26‐fold on day 7 (*p* < .0001; Figure 6a,b), whereas the ratio of phosphorylated Akt (p‐Akt) to total Akt protein was significantly downregulated by 0.39‐fold on day 7 (*p* < .01; Figure 6c). Moreover, muscle atrogin‐1 mRNA expression was significantly upregulated in AKI mice by 2.89‐fold on day 1 (*p* < .01) and 6.61‐fold on day 7 (*p* < .0001; Figure 7a,b). No differences were observed in the LC3 II/I ratio, a marker of autophagy activation, between the AKI and sham‐operated groups (Figure S4); however, mRNA expression of peroxisome proliferator‐activated receptor gamma coactivator 1α (PGC‐1α) was decreased by 0.59‐fold on day 1 in the AKI group (*p* < .05; Figure 7c).

### 3.5 Effect of exercise and BCAA supplementation on muscle‐related molecular markers

Combined exercise and BCAA supplementation did not affect myostatin mRNA expression on day 1, while significantly decreasing its expression on day 7 (*p* < .01; Figure 6a,b) and restoring p‐Akt abundance compared to the sham‐operated group (Figure 6c; Figure S3). Moreover, the combined treatment did not alter muscle atrogin‐1 mRNA expression on day 1, however, significantly decreased its expression on day 7 (*p* < .0001, Figure 7a,b) and restored PGC‐1α mRNA expression compared to the sham‐operated group on day 1 (Figure 7c). No differences were observed in the LC3 II/I protein ratio between the three experimental groups (Figure S4). Therefore, exercise and BCAA supplementation for 7 days prevents AKI‐induced upregulation of myostatin and downregulation of atrogin‐1, respectively.

## 4 DISCUSSION

**Figure 8:**
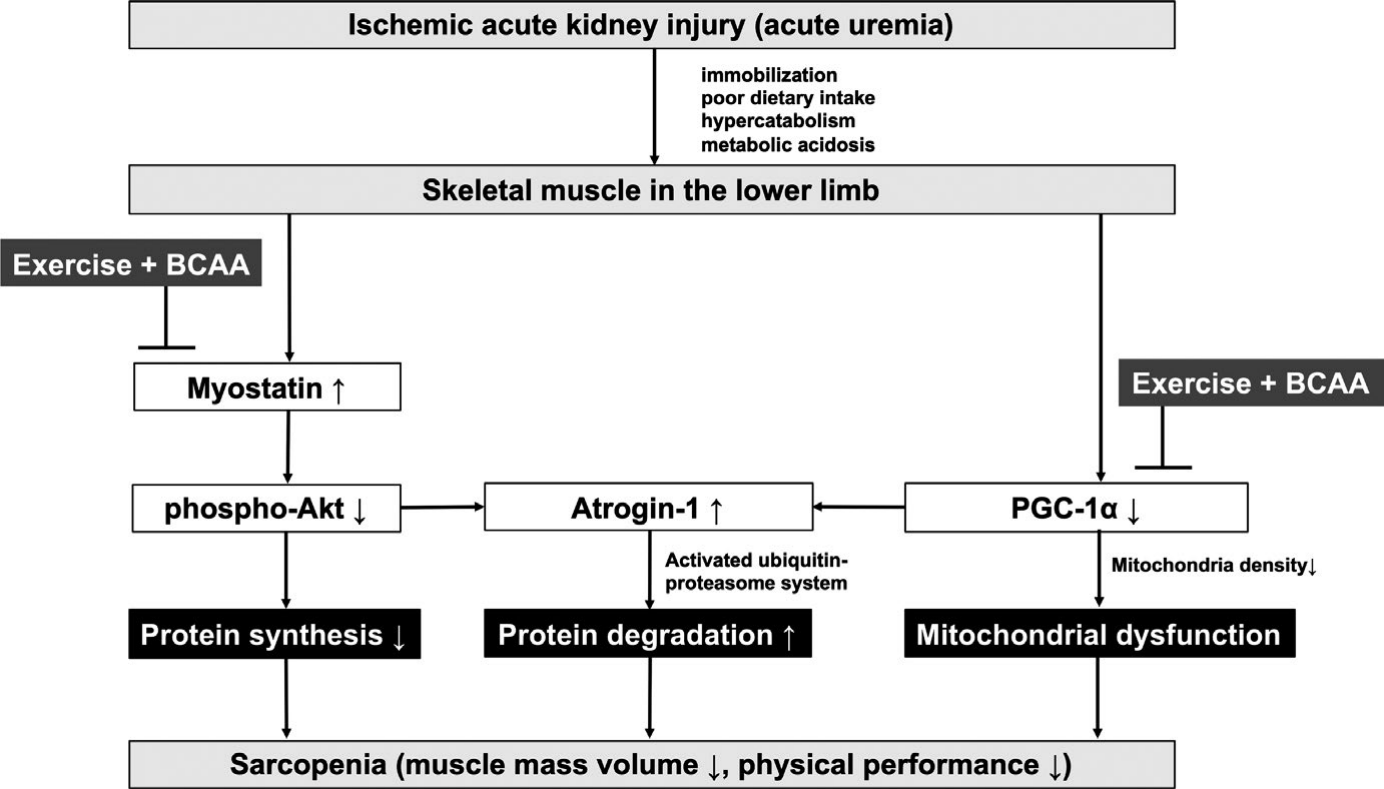
Effect of exercise and BCAA treatment on AKI‐related muscle wasting. BCAA, branched‐chain amino acids

This study demonstrated that ischemic AKI significantly decreases muscle mass, myofiber CSA, interfibrillar mitochondrial density, and maximal running time after 7 days. Meanwhile, regular exercise for 30 min per day and oral BCAA supplementation for 7 days prevents AKI‐related muscle wasting, suppresses myostatin and atrogin‐1 mRNA expression, increases the p‐Akt/Akt ratio, and restores PGC‐1α mRNA to baseline levels. These findings suggest that ischemic AKI causes muscle wasting by impairing protein synthesis, activating protein degradation via the ubiquitin‐proteasome system (UPS), and causing mitochondrial dysfunction, all of which were restored by regular treadmill exercise and BCAA supplementation (Figure 8).

### 4.1 Effect of anorexia on AKI‐related muscle wasting

Gentamicin‐induced AKI has been reported to cause extensor digitorum longus muscle wasting after 5 days (Aniort et al., 2016); however, this study did not collect dietary intake data after exposure. Since skeletal muscle wasting is a debilitating response to poor eating, we conducted strict pair feeding for 7 days, after which BW, muscle mass, myofiber CSA, and maximal running time were significantly decreased in AKI mice despite consuming the same amount of food and water for 7 days. A significant positive relationship was also observed between tGFR and tibialis anterior muscle wet weight on day 7 (Figure S1), indicating that AKI mice are likely to exhibit muscle wasting caused by acute uremia‐mediated protein metabolism dysregulation.

### 4.2 Mechanisms of AKI‐related muscle wasting

Muscle wasting may be caused by suppressed protein synthesis and enhanced protein degradation. Myostatin is a member of the transforming growth factor β (TGF‐β) family that is predominantly expressed in skeletal muscles and suppresses their growth (Wang & Mitch, 2014). After myostatin binds to its receptor, myostatin inactivates Akt by increasing phosphatidylinositol 3,4,5‐trisphosphate 3 phosphatase and dual‐specificity protein phosphatase (PTEN) expression (Wang & Mitch, 2014), resulting in dephosphorylation of the Forkhead box O (FoxO) transcription factor family and causing muscle wasting (Sandri et al., 2004). Dephosphorylated FoxO transcription factors enter the nucleus to increase the expression of the E3 ubiquitin‐ligases TRIM63 (also known as MuRF1) and F‐box only protein 32 (MAFbx, also known as atrogin‐1), which stimulate UPS‐mediated muscle protein degradation (Gomes, Lecker, Jagoe, Navon, & Goldberg, 2001). In CKD mice, Akt overexpression or myostatin inhibition have been shown to prevent muscle wasting by suppressing protein degradation and improving satellite cell function (Hanatani et al., 2014; Zhang et al., 2011). Moreover, inflammatory cytokines (Zhang et al., 2009), metabolic acidosis (Bailey et al., 1996), and uremic toxins (Enoki et al., 2017) also activate proteolysis via the UPS. Although a previous study (Price et al., 1998) demonstrated that acute uremia caused by bilateral ureteral ligation increased BCAA catabolism in the gastrocnemius muscle, few studies have examined the molecular mechanisms of muscle protein synthesis and degradation in an AKI model. Here, we found that AKI significantly upregulates myostatin and atrogin‐1 mRNA expression, while significantly reducing Akt phosphorylation, indicating that myostatin overexpression may mediate AKI‐related muscle wasting by suppressing Akt phosphorylation and increasing atrogin‐1 mRNA expression.

### 4.3 Role of kidney‐muscle crosstalk in AKI‐related muscle wasting

Ischemic AKI causes distinct damage to organs including the lungs, liver, and intestines (Doi et al., 2014; Golab et al., 2009; Nakazawa et al., 2017; Park et al., 2011). Skeletal myocytes can recognize pathogen‐associated molecules via toll‐like receptors (TLRs) 2 and 4 to initiate an interleukin (IL)‐6 transcriptional response (De Moraes et al., 2018; Frost, Nystrom, & Lang, 2006). Therefore, we induced a 35‐min IRI in the left kidney with an intact right kidney to examine the role of organ crosstalk between damaged kidneys and skeletal muscle under non‐uremic conditions. Although a transient increase in muscle atrogin‐1 mRNA expression was observed on day 1, muscle weight did not change after pair feeding (Figure S2a–d), suggesting that crosstalk between the acutely injured kidney and muscle plays a minor role in the development of muscle wasting.

### 4.4 Potential mechanisms of exercise and BCAA supplementation in AKI‐related muscle wasting

#### 4.4.1 Exercise

Regular exercise exerts a positive effect on muscle mass in CKD by enhancing protein synthesis and suppressing proteolysis via UPS activation (Bacurau et al., 2016; De Moraes et al., 2018; Sakai et al., 2017; Yoshida et al., 2017). For instance, exercise has been found to increase PGC‐1α mRNA expression, a master regulator of mitochondrial biosynthesis and energy metabolism (Cantó & Auwerx, 2009; Wu et al., 1999). PGC‐1α is also involved in the shift from group IIb to group I and IIa muscle fibers, thereby improving exercise tolerance by enhancing mitochondrial biosynthesis (Lin et al., 2002). PGC‐1 has also been shown to protect skeletal muscle wasting by suppressing the action of FoxO3 and the expression of atrogin‐1 and MuRF‐1 in rats with chronic renal failure (Sandri et al., 2006). Moreover, 8‐weeks of progressive resistance training was found to suppress myostatin mRNA expression and increase p‐Akt abundance by twofold in vastus lateralis skeletal muscle biopsies from patients with CKD (Watson et al., 2017). In this study, the combined treatment restored PGC‐1α mRNA expression by day 1 and suppressed upregulated myostatin and atrogin‐1 mRNA expression by day 7. Thus, regular exercise prevented muscle wasting by increasing PGC‐1 mRNA expression during the early phase of ischemic AKI and suppressing myostatin and atrogin‐1 mRNA overexpression during the later phases.

#### 4.4.2 BCAA supplementation

BCAA may increase muscle protein synthesis and inhibit proteolysis by suppressing the UPS; for instance, leucine supplementation has been shown to minimize atrogin‐1 overexpression in immobilized rats (Baptista et al., 2013). Since BCAA may cause glomerular hyperfiltration and aggravate renal prognosis (Pillai et al., 2019), few studies have examined the effect of BCAA alone on muscle in renal injury models. Yoshida et al. reported (Yoshida et al., 2017) that BCAA administration alone was ineffective against CKD‐induced muscle wasting in 5/6 nephrectomized rats, but had a synergistic effect with exercise without the progression in CKD. Similarly, in a human study, BCAA, when used in combination with exercise, enhanced phosphorylation of mammalian target of rapamycin (mTOR) complex 1 and ribosomal protein S6 kinase (S6K) beta‐1, and decreased atrogin‐1 and MuRF‐1 mRNA levels, in vastus lateralis muscle (Borgenvik, Apró, & Blomstrand, 2012). Rabkin et al. reported (McIntire, Chen, Sood, & Rabkin, 2014) that acute uremia induces resistance to leucine‐stimulated anabolic signaling based on the relative changes in the phosphorylation of mTOR and downstream proteins such as eukaryotic translation initiation factor 4E‐binding protein 1 (4E‐BP1) and S6K, 44 hr following bilateral ureteral obstruction.

In their report, rats were acutely administered leucine in water at a concentration of 1.35 g per kg body weight, an amount approximating a rat's typical daily intake, at 10 min immediately prior to euthanasia. They could not detect muscle wasting in their AKI rats compared with sham‐operated pair‐fed controls, probably due to the short‐term duration of uremia (44 hr). In the present study, we observed the changes of skeletal muscle volume and physical function until 7 days after ischemic AKI, and confirmed that ischemic AKI induced loss of muscle mass volume and physical function when compared with sham‐operated mice, despite strict pair feeding for 7 days. In addition, the combined treatment of exercise and BCAA treatment for 7 days reduced AKI‐induced muscle wasting morphologically and functionally. Although there are some differences between our results and those previously reported with renal injury models and or when using a different BCAA administration period, at present, we have not conducted BCAA single administration experiments so are unable to make a direct comparison.

### 4.5 Effect of exercise and BCAA supplementation in AKI

In this study, regular exercise and oral BCAA administration did not affect the decline of tGFR or tubular damage until day 7. However, exercise has been shown to reduce TGF‐β and fibrosis in CKD (Wang & Mitch, 2014). In addition, Higa et al. reported (Oliveira et al., 2017) that moderate exercise training for 30 days mitigates AKI‐induced oxidative stress and TGF‐β upregulation in the kidney during gentamicin‐induced AKI. Thus, long‐term exercise may improve ischemic AKI recovery by facilitating recruitment of proximal tubular cells to terminate regenerative processes.

### 4.6 Clinical application to AKI patients

In clinical practice, AKI patients are debilitated and it may be difficult for them to perform treadmill training. However, Hickmann et al. reported (Hickmann et al., 2018) that early physical therapy (twice daily sessions of both manual mobilization and 30‐min passive/active cycling) within the first 72 hr following the onset of septic shock regulates catabolic signals and preserves skeletal muscle mass in adult patients admitted for septic shock. Future clinical trials will be needed to ascertain the effect of exercise and BCAA treatment in the early phase of AKI on muscle wasting, especially in ICU patients.

## 5 CONCLUSIONS

This study demonstrated that acute uremia induced by experimental ischemic AKI causes skeletal muscle wasting for 7 days despite careful pair feeding. However, the combination of regular exercise and BCAA supplementation for 7 days prevented AKI‐related muscle wasting by suppressing myostatin and atrogin‐1 mRNA upregulation, and restoring Akt phosphorylation and the level of PGC‐1α, without causing further kidney dysfunction.

## CONFLICT OF INTEREST

No conflicts of interest, financial or otherwise, are declared by the authors.

## AUTHOR CONTRIBUTIONS

S.N., A.K., T.F., and H.Y. conceived and designed research; S.N. performed experiments; S.N., A.K., and T.F. analyzed data; S.N., A.K., S.I., T.F, N.O., H.M., and H.Y. interpreted results of experiments; S.N. and A.K. prepared figures; S.N. and A.K. drafted manuscript; S.N. and A.K. edited and revised manuscript; S.N., A.K., S.I., T.F, N.O., H.M., and H.Y. approved final version of manuscript.

## Supporting information



Appendix S1
