# Supplementary material for: Regular exercise and branched‐chain amino acids prevent ischemic acute kidney injury‐related muscle wasting in mice
Source: Physiol Rep. 2020 Aug 26;8(16):e14557. doi: 10.14814/phy2.14557 (PMC7448801; doi:10.14814/phy2.14557)
Supplement: Supplementary file 1 — Appendix S1 [file PHY2-8-e14557-s001.docx]

***Supplemental Materials***

*Supplemental Table. 1. Primers used for real-time qRT-PCR*.

| Gene | Forward Primer Sequence | Reverse Primer Sequence | bp |
| --- | --- | --- | --- |
| β-Actin | 5ʹ-GTGACGTTGACATCCGTAAAG-3ʹ | 5ʹ-GCCGGACTCATCGTACTCC-3ʹ | 245 |
| Atrogin-1 | 5ʹ-GCAAACACTGCCACATTCTCTC-3ʹ | 5ʹ-CTTGAGGGGAAAGTGAGACG-3ʹ | 93 |
| Myostatin | 5ʹ-CTGTAACCTTCCCAGGACCA-3ʹ | 5ʹ-TCTTTTGGGTGCGATAATCC-3ʹ | 197 |
| PGC-1α | 5ʹ-AAGTGGTGTAGCGACCAATCG-3ʹ | 5ʹ-AATGAGGGCAATCCGTCTTCA-3ʹ | 161 |

*Supplemental Table. 2. Protocol of the exercise tolerance test*

| Stage | Time(min) | Speed(m/min) | Duration(min) |
| --- | --- | --- | --- |
| 1 | 0–5 | 10 | 5 |
| 2 | 5–7 | 12 | 5 |
| 3+ | +2 | +2 | 2 |

*Inclined at 0 ° on all stages


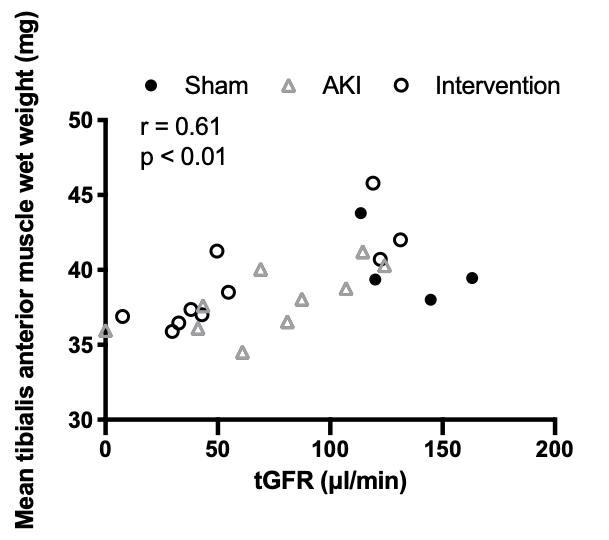


**Supplemental Fig. 1 Relationship between tibialis anterior muscle wet weight and tGFR** Muscle weight and tGFR displayed a significant positive correlation (*r* = 0.61, *p* < 0.01). AKI, acute kidney injury; tGFR, transcutaneous glomerular filtration rate


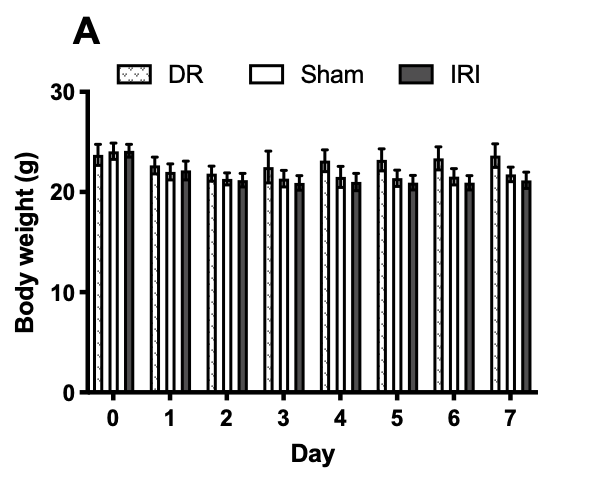

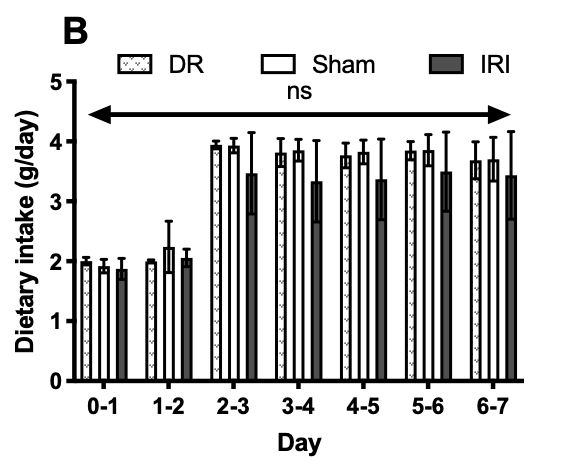

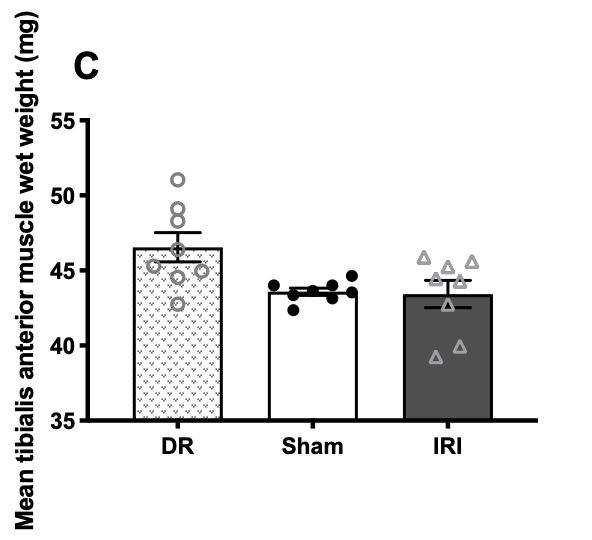

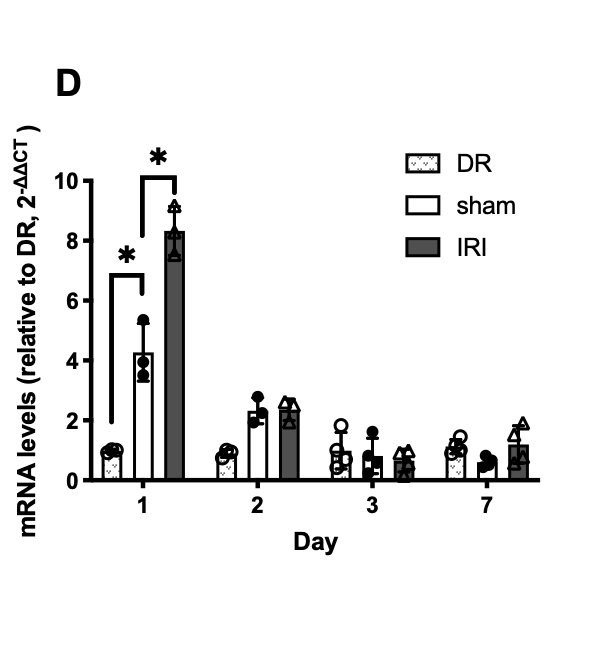


**Supplemental Fig. 2 Effect of unilateral ischemia-reperfusion injury on BW, muscle weight, dietary intake, and atrogin-1 mRNA expression in tibialis anterior muscle** Ischemia-reperfusion injury (IRI) of the left kidney for 35 min did not increase serum creatinine levels or affect BW (A) and muscle mass weight (C) between the pair-fed non-operated, sham-operated and left IRI groups during the 7 days after insult (B). Atrogin-1 mRNA expression increased transiently on day 1 but returned to baseline levels on days 2 and 7 (D). Data represent the mean ± standard deviation (A-C: *n* = 8, D: *n* = 3). Significant differences between DR group and the other groups: **p* < 0.05. DR, dietary restriction; IRI, ischemia-reperfusion.


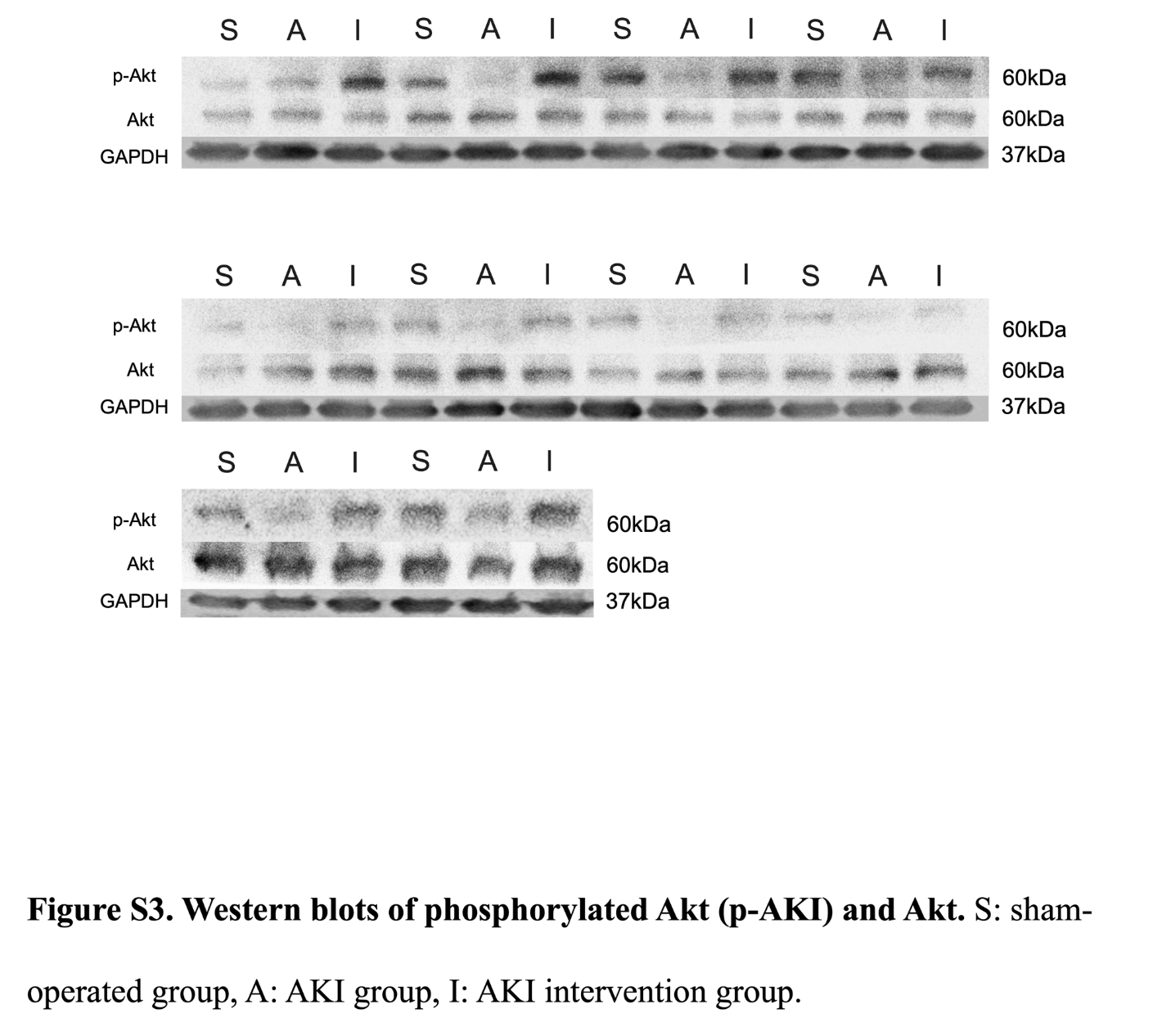


**Supplemental Fig. 3 Western blots of phosphorylated Akt (p-AKI) and Akt** S, sham-operated group; A, AKI group; I, AKI intervention group


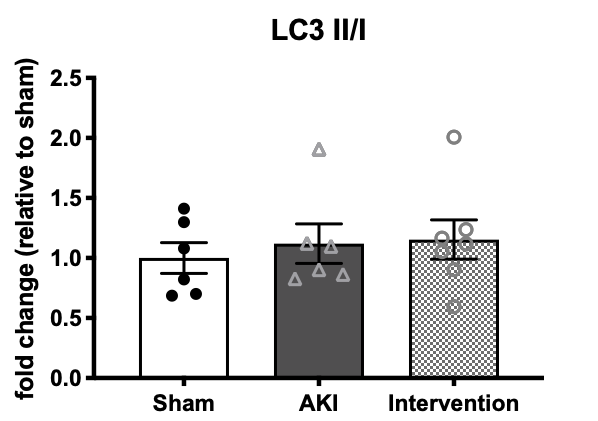


**Supplemental Fig. 4 Changes in the muscle LC3-II/I ratio on day 7 after AKI** No differences were observed in the LC3 II/I ratio of the three groups. AKI, acute kidney injury
